# A typical case of flood syndrome

**DOI:** 10.11604/pamj.2023.45.159.40944

**Published:** 2023-08-15

**Authors:** Anjana Ledwani, Babaji Ghewade

**Affiliations:** 1 Department of Respiratory Medicine, Jawaharlal Nehru Medical College, Datta Meghe Institute of Higher Education and Research, Sawangi (Meghe), Wardha, Maharashtra, India

**Keywords:** Flood syndrome, ascites, umbilical hernia, spontaneous paracentesis

## Image in medicine

A 45-year-old male patient, with a case of cirrhosis with portal hypertension, massive ascites, and a large umbilical hernia with ulceration presented to the hospital with sudden rupture of the hernia with drainage of ascitic fluid from the abdomen. The spontaneous rupture of an umbilical hernia with a sudden gush of fluid is known as flood syndrome. This syndrome is an uncommon consequence of chronic ascites and end-stage liver disease and is named for the surge of fluid that follows the spontaneous rupture of an umbilical hernia. An abrupt rise in intra-abdominal pressure that is accompanied by coughing, vomiting, straining, or getting up from a chair may be followed by rupture. Umbilical hernia rupture is typically preceded by the development of cutaneous ulcerations (80% of the time). The development of cellulitis, peritonitis, and sepsis are complications of umbilical hernia rupture, as well as bowel imprisonment, and hypotension brought on by large-volume spontaneous paracentesis. Urgent surgical referral should be made if an umbilical hernia develops an ulcer or necrosis over it as this is a serious sign that indicates an impending rupture. In the present case, the patient’s condition worsened during the course of the hospital as he developed high-grade fever and hypotension, his blood parameters showed an elevated white blood cell count (WBC) of 18,000/cu.mm and he was started on intravenous antibiotics and vasopressors. The patient was taken up for Transjugular Intrahepatic Portosystemic Shunt (TIPS) after which the patient became hemodynamically stable. Abdominal hernia repair was postponed owing to his poor medical condition. The patient improved symptomatically upon the treatment of his massive ascites and was discharged in stable condition with the advice of regular follow-up.

**Figure 1:**
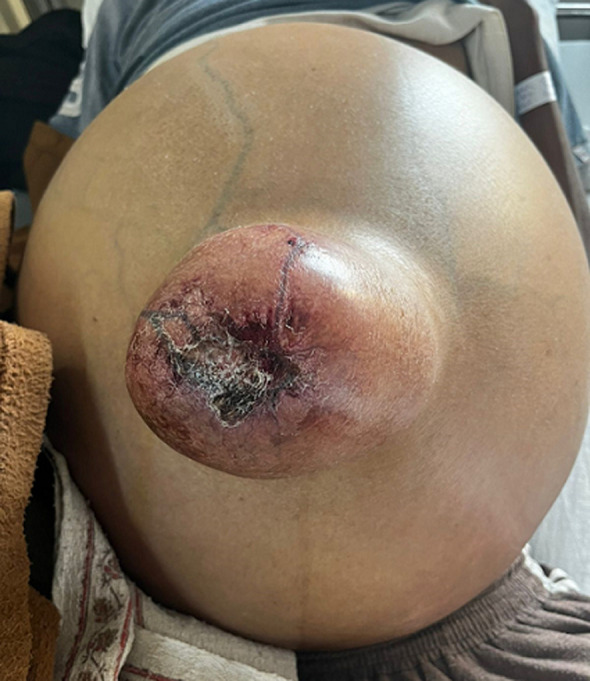
ulcerated umbilical hernia in a case of massive ascites

